# The Parliamentary Inquiry and Popular Notions Concerning the Treatment of Lunatics

**Published:** 1860-01-01

**Authors:** 


### ART. III.—THE PARLIAMENTARY INQUIRY AND POPULAR NOTIONS CONCERNING THE TREATMENT OF LUNATICS.

A RECENT writer* has well and truly said that, "Falsehood is so easy, truth so difficult. The pencil is conscious of a delightful facility in drawing a griffin—the longer the claws, and the longer the wings, the better; but that marvellous facility which we took for genius, is apt to forsake us when we want to draw a real unexaggerated lion. Examine your words well, and you will find that even when you have no motive to be false, it is a very hard thing to say the exact truth—even about your own immediate feelings—much harder than to say something fine which is *not* the exact truth."

No apter illustration of the accuracy of these remarks could be found than that which is afforded by much of the evidence laid before the Select Committees which sat in the last and preceding sessions of Parliament to inquire into the operation of the laws affecting the care and treatment of lunatics. Six months ago we reviewed the evidence laid before the Committee of 1858–59; we have now before us the evidence tendered to the Committee of last session.† In our notice of the proceedings of the first Committee our attention was chiefly occupied with the evidence given by the Right Hon. the Earl of Shaftesbury, the Chairman of the Lunacy Commission. This evidence claimed particular attention at our hands, not only from the official position which his lordship occupies, but also from the extraordinary character of many of the opinions which he expressed, and statements that he made. It will be well to recal here to memory one or two of the most notable points of his lordship's evidence.

On the question which of all others, in reference to lunatics,

* George Eliot.—*Adam Bede.*

† Report from the Select Committee on Lunatics—Blue Book. Ordered to be printed, 5th August, 1859.

had most occupied the public attention, the regulations respecting the admission of lunatics into private asylums, his lordship on behalf of the Commissioners in Lunacy suggested a modification of the existing law—not that he himself,* or, indeed, the Commissioners either, as it was implied, saw any necessity for, or believed that any good would arise from, altering the present regulations, but "*only as an expedient with a view of satisfying the public feeling, and not with any hope that it would really be effective.*"†

Again, on another question of very great interest for the public, in so far as it affected its confidence in present or future legislative arrangements, his lordship's opinions were characterized by singular inconsistencies and contradictions. He emitted "(1) an assertion of the comparative worthlessness of the opinion of most medical men on cases of lunacy; (2) an expression of "firm belief" that the opinion of a sensible layman on such cases is of greater value than that of a medical man; (3) an assertion that the opinion of a magistrate is valueless, from his knowing nothing whatever about the matter; (4) another assertion that the opinion of a clergyman is even of less value than that of a medical man; (5) a statement that an opinion depends for its value on the experience of the person giving it; and, to crown all, (6) an approval of a suggestion by which the bulk of the most experienced practitioners in lunacy in the kingdom would be debarred from altering an opinion at all in the form in which a medical opinion on lunacy is of the greatest importance—to wit, when expressed in a certificate of insanity."‡

With regard to insanity itself we may remind our readers that his lordship's views were so peculiarly vague§ that they would have been amusing were it not for the speculative dogmatism which he developed from them, and which derived from his position no small amount of importance. We need only recal one more point, to wit, the exaggerated assertions of his lordship respecting the "utterly abominable and indefensible|| system of private asylums"—assertions which he endeavoured to support mainly, (1) by facts derived from the state of these institutions *before* 1846! since which year the powers of the Commissioners have come fully and effectively into operation; and (2) by *speculations* alone as to the existing state of things.

We protested against the unfounded, nay, entirely gratuitous, character of the surmises upon which Lord Shaftesbury built up his evidence concerning private asylums, and expressed the opinion that if such surmises, or such a system of reasoning,

* Report, 1858–59, Query 823.

† Report, 1858–59, Query 81.

‡ See Vol. xii. of this Journal, p. 382.

§ Reports, 1858–59, Queries 193, 194.

|| Report, 1858–59. Query 101.

was to prompt the action of the Lunacy Commission, or influence legislation, it must necessarily debar all right-thinking medical men from giving attention to the care and treatment of private lunatics, and must place these at the mercy of those who were actuated by mercenary considerations alone.

We are glad to observe that one of the Commissioners of Lunacy, W. G. Campbell, Esq., has in his evidence before the late Committee, expressed a species of protest against the speculative character of Lord Shaftesbury's statements on this subject. At the commencement of his evidence Mr. Campbell made the following remarks:—

"I wish to call the attention of the Committee to a point which seems to me to have been forgotten by some of the witnesses, and that is, that asylums are places for the cure and treatment of the insane as well as places of detention, and I think that great care should be used when we surround asylums with so many safeguards, that we do not degrade them, and also those who keep them; I speak of licensed houses; I am fully aware of the defective and very unadvisable arrangement of having a licensed house for the detention of insane persons, because the treatment of the insane involves the detention of the person. The fact of a person receiving another for profit, and having the power to deprive him of his liberty, is, I think, a most objectionable arrangement; but the question is whether that arrangement can be got rid of, and whether all persons can by law be forced into chartered hospitals or public asylums; *I think, if that cannot be accomplished, that it is desirable to try and induce persons of the highest character only to take licensed houses and receive patients. I am afraid that by degrading them and showing such extreme suspicion of all these persons, by treating every one who has the care of an asylum or licensed house, as a person who is* primâ facie *a man who would take advantage of his patients and deprive them of their liberty for profit, we shall be doing an injury to the cause.*"*

We welcome warmly the protest against Lord Shaftesbury's suspicions which is contained in these observations, but we would ask the Commissioners in Lunacy if it never occurred to them that the "principle of profit" (as they phrase it) has, in reference to private asylums, a good as well as an evil side, just as it has to everything else in the business of life. If the Commissioners will look about them, they will see among the proprietors of private asylums abroad and at home many men who, having given themselves up to this specific and legitimate mode of obtaining a livelihood, have made and still make it available for the advancement of our knowledge in all that relates to the care and treatment of the direst disease which affects man—men to whom the world is indebted for almost all the knowledge that it has of

* Report 1859, Query 380. The subsequent references to Queries will apply to this Report, unless otherwise stated.

late received concerning the best care of lunatics—men, the fag-ends of whose notions constitute the stock-in-trade of the *quasi*-lunatics'-friends philanthropists of the present day. Would the experience, which these medical men have lavished upon the public, have been acquired in a public asylum alone? Has any public asylum hitherto afforded the means for a full and comprehensive study of insanity among all classes of society? Has it not been from the combined information obtained both in public and private lunacy practice, that the great advancement in the care of lunatics which we now rejoice in has been secured? To speak of the "principle of profit" as an evil *per se*, is simply absurd. It has been formerly, and may be in some instances now, unrighteously exercised in private asylums; but it is the man, not the principle, which is in fault, and the law rightly provides a remedy for this by assigning to the Commissioners as one duty among others, that of ascertaining the fitness of all persons who are or who wish to become proprietors of private asylums. Hence with the Commissioners rests the responsibility of, as Mr. Campbell would put it, "inducing persons of the highest character only to take licensed houses."

We have already seen that Lord Shaftesbury suggested legislative interference upon one point of lunacy law, not because it was required in reality, but because it was expedient on account of popular feeling. Mr. Campbell has a remark about popular feeling which is well worthy of note. Speaking of private asylums, he says, "The great objection to them is the impression that they produce upon the mind of the public that the patients in them are detained for profit, and that, therefore, persons who are not of unsound mind are sometimes detained improperly as a source of profit."* Mr. Campbell tells us† that he does not consider it possible to abolish the system of private asylums by law, and that that being so, all the Legislature can do is to subject them to rigid supervision, adding, "and they are subject to rigid supervision at present."‡ Lord Shaftesbury told us that the notion of improper admissions or detentions was essentially wrong,§ and left it to be implied that such occurrences could only take place at rare intervals and under unusual circumstances. The public idea, therefore, can have no sufficient foundation in fact, but must be a corollary deduced from the theoretical idea of the "principle of profit,"—as Mr. Campbell puts it. Be it so. But the facts, according to Lord Shaftesbury, are not such as to warrant a "great objection;" and Mr. Campbell says nothing to the contrary; hence the theory must be wrong, and the popular notion quite astray. What then must be done with it? Oh! the course is plain before us; we must turn over our books, and lay

* Query 612. † Query 615. ‡ Query 614. § Report 1858-59. Qy. 185.

before the public such details as will at once allay its fears, and convince it that any instances of unjust detention or committal which may have come before it of late have and probably would have been unavoidable under any system of management. Have the Commissioners done this? Turn to Lord Shaftesbury's evidence, and you will see that he has, upon suspicion alone, expressed opinions which surpass in gravity the worst fears of an alarmed press; that upon merely speculative grounds, unsupported by hardly a particle of evidence, his lordship has said more to depreciate the moral and general character of the medical profession practising in lunacy—of those men to whom alone, as his lordship himself states, it is best to give the charge of the lunatic*—than has been said at any one time by any individual of station for several years. More, he has spoken generally of the private asylums of this day much as if they were the lunatic hells of a former day. He, and by implication the Lunacy Commissioners, have said all that could be said which might confirm a popular notion that cannot be substantiated in fact; they have brought the weight of their authority to establish the notion; and yet, strangest of culminations! the public feeling is paraded by them as a reason for legislative interference!

Thus much for the official representatives of public feeling in the inquisition; now for a few examples of the lay representatives. The gentlemen who come within this category were Mr. John Thomas Perceval and Admiral Saumarez.

The former gentleman is the honorary secretary to the alleged Lunatics' Friend Society, and considers himself the "attorney-general of her Majesty's madmen." Contrast for a moment the professed interest he takes in reference to lunatics, and the actual. The question was put to him, "I think you have heard a good deal of the evidence that has been given before this Committee?" He replied, "Not a great deal; I am rather dull of hearing; but I have had a copy of the report, and I have read a great deal of the evidence; all of Lord Shaftesbury's evidence, and a great deal of Mr. Bolden's and Mr. Everest's evidence, and some of Dr. Gaskell's evidence, and other portions of the evidence."† Truly one would have thought that the perusal of a Blue Book containing only three hundred and seventy-eight pages, including the index, would not have taxed too much the time and patience of a gentleman arrogating to himself so lofty and philanthropical a position. Surely the evidence of Dr. Conolly, Dr. Sutherland, Dr. James Bright, or Dr. Hood; Messrs. Wilde, Barlow, Purnell,

* Report 1858-59; Queries 82, 87, 94, &c. † Query 159.

Cottrell, or Woodward; Sir Joshua Jebb, Sir A. Spearman, and Sir G. Robinson, was at least worthy of perusal. But some persons have an instinctive knowledge of things; we shall see in the context if this be Mr. Perceval's case.

Mr. Perceval has certain peculiar views which he very rightly places in the fore-front of his evidence, for they serve well as a gauge of its quality. He says:—

"I would add my peculiar views, and what has been perplexed in some manner, but still has been my guiding star, and I may say my chief principle in interfering in this matter. It has been a motive of piety as regards the Founder and Establisher of our own faith. It appears to me that no Christian nation should leave a loophole open for any preacher or teacher of any kind to be confined as insane when it is recollected that the Founder of our faith and his apostles were accused of being possessed with demons and being madmen.

"162. These are abstract and rather metaphysical questions. What the Committee rather desire to obtain from you is such suggestions as you could make as would prevent a person being confined as a lunatic, when only under an hallucination, or in fact in perfect health. How would you meet the dangers of the system?—I would say that the same observations which I have made in a religious sense apply also to men of science, to men of political or any other science. We know that it is chronicled in history, that Galileo was supposed to be out of his mind for simply teaching that the earth moved round the sun. Mr. Forster, the original inventor of railroads, before Stephenson took them up, went about from England to Belgium, and from Belgium to France, and he was looked upon as half an insane person. Therefore I think that the Government of any country, without any regard especially to religion, should be particularly jealous of any man, or of any 'original mind,' as the French describe it,—we call it 'eccentric,'—being shut up on a false charge of insanity. It appears to me, said a young Frenchman to a friend of mine, that you may put any man who is a little original in an asylum; and it is the case on account of the lax way of legislating on the subject. And it must be remembered that they have been men of original minds who have changed the destinies of nations and the destinies of the world."

What a charming delicacy in the young Frenchman's remark, and how pretty the conceit, "eccentric,"—"original mind." We commend this notion to the distinguished author of the *Thesaurus of English Words and Phrases.*

"All that is conceded to you," was the polite expression with which the chairman of the Committee checked any further exposition of Mr. Perceval's abstract notions; and so to business.

As might be anticipated from the premises set forth by Mr. Perceval, the class of patients which he would admit to asylums would be somewhat restricted. In reference to Lord Shaftesbury's remark, that no case is admitted into an asylum without some plausible ground for confinement, Mr. Perceval said, "I do not allow in these cases of any plausibilities. I want plain matter-of-fact to be ascertained, whether a patient is a dangerous person to himself or to others. Whether he is a dangerous person to himself directly—I mean in a suicidal sense, or indirectly by being exposed through weakness to robbery, or that they might be guilty of acts of robbery, or petty theft, or libel, which might bring punishment upon them. These, I think, are the only grounds upon which persons ought to be confined in an asylum."*

It will be seen that Mr. Perceval's plain matter-of-fact is of a very imaginative character, and quite consistent in its coherency with his abstract notions. It is evident that he has given as little attention to the cases of even very manifest insanity usually confined in asylums, as to the preliminary stages of the inquiry upon which he gave evidence.

As an example of Mr. Perceval's method of observation and reasoning take the following:—

"229. Are you not aware that it is the general opinion of persons who have dealt with lunatic patients, that, from the nature of the disorder, private conversations between ministers of religion and the patients are very injurious, although, in many instances, they may be very beneficial, so that the superintendent must exercise some discretion in such matters?—*I am not aware of that.* I am aware that there is a great prejudice to that effect; but whether it is the case or not, I am certainly not aware. On the contrary, we have heard from the evidence, and from the Commissioners in Lunacy themselves, that great benefit has been derived from it. But, at the same time, I have no doubt that there are cases in which a clergyman might interfere very injudiciously. Certainly a latitude should be given to the superintendent; so long as the present system exists, you must leave it to him to say whether a patient is fit to see a clergyman or not.

"230. I meant to draw you to the conclusion, that in all these cases it must be left to the discretion of the superintendent?—At present it must be so; I have remonstrated against that state of things; I think it contrary to principle that the spiritual should be put under subjection to the physical."

As a further example of the value of Mr. Perceval's evidence, we find him, in answer to Query 290, describing with vigour the brutal treatment of patients in Bethlem and another asylum in 1853. The chairman of the Committee asked—"Is there any reason to suppose that there is any asylum in which that practice is continued now?" Mr. Perceval replied, "*That I cannot tell; I do not think that we have any evidence for it, but I should not*

* Query 373.

*be at all surprised to find it so again.*"* Just so; what have I to do with facts? I am concerned solely with my own ideas.

Let us glance for a moment or two at this gentleman's style of assertion. For example:—

"There is a gentleman of the name of Mr. ——, who has known many cases where persons have been guilty of criminal acts in the full possession of their senses, and they have been enabled to escape the consequence of their acts by being confined as insane, under the certificates of medical men.

"245. Mr. *Henley*. They simulate insanity to escape the consequences of their criminal acts?—Yes, under the direction of their friends."

Not a single example was mentioned by Mr. Perceval in confirmation of this—assertion (we had better perhaps omit placing the correct adjective which should precede this word). We have not the least doubt that he could not recount one solitary instance of the kind named of late, and probably even of former years.

Again:—

"I wish to notice one part of the evidence that I gave as to the propriety of the friends and relations of the patients having power to visit them at all reasonable hours. The Honourable Member, Mr. Henley, objects to that, and I think with some good reason, and said that he thought even in small asylums it would be better that there should be fixed hours for visiting them. One of my friends, who has suffered under this law, said to me, 'I am sorry that you gave in to the Honourable Member in this respect, because if they are visited at fixed hours, the keeper of the asylum knows when they may come, and he may drug the patients for the occasion.' *This is, certainly, I am sorry to say, a possibility in some asylums, and perhaps in particular cases.*"

And so Mr. Perceval goes on from surmise to surmise with a felicity of imagination, and an indifference to fact, which are verily astounding.

To proceed:—

"282. What is the next point to which you wish to call the attention of the Committee?—The next point is as to the existence of cruelty in asylums, and I wish to point out what I consider the only manner of thoroughly preventing it. With regard to that question, I think it is a doubt in some minds in the Committee whether cruelty does take place in asylums generally, but in private or public asylums particularly. With regard to that impression, I have no hesitation in saying, that I believe generally, as respects all asylums, there is not one of them in which cases of cruelty and atrocious violence do not continually occur.

"283. Do you apply that remark to public asylums as well as to private asylums?—Yes; that is my impression. There is an old saying, that

* Query 297.

one swallow does not make a spring; I know that; but when you see a swallow or a martin in the month of May, it is natural to look about and think that you will find a great many. Then I apply that to the system in these asylums; one case of cruelty in a private asylum would not prove anything against the whole of the asylums. But if you can trace it to the system of the law, and to the want of inspection and proper protection to the patients, and to the laws governing all asylums as well as that particular asylum, then you have a right to say, that by analogy it is not at all unlikely that the same system prevails in other asylums, as you have detected it in one instance. *This was the impression on my mind when I first knew* of the atrocious cruelties which could be practised in private asylums, and that not only from personal violence, but from what you may call a normal state of ignorance and cruel treatment."

The *naïveté* of this confession respecting the origin and foundation of his belief on this head in suspicion alone is truly exquisite.

Again, speaking further of cruelties exercised in asylums, Mr. Perceval said:—

"What I call cruelty in an asylum, and reckless cruelty, is, where the patients are very often, not from any fault of their own, provoked and tantalized, and worked up, as it is called, by an allusion to their own infirmities, and where they are all of a sudden seized and thrown with any amount of reckless violence on a stone floor, or across an iron bedstead, and they are then kneaded with the knee on the chest, at the risk of breaking their ribs; then they are seized by the throat until the eyes start out of the sockets, and the blood comes out of the mouth. They are also taken by the neckcloth and their heads thumped against the floor or banged against the wall. That is the normal treatment, I fear, of the patients in the refractory wards generally throughout the country.

"310. You say that that is the normal treatment. There has been a great deal of evidence given, that the normal treatment of lunatics and people unsound in their minds, some years ago, was entirely different from the normal treatment going on now?—Yes.

"311. I think you had better keep the two classes of cases distinct. Lord Shaftesbury stated that he believed there were great cases of cruelty going on in private asylums, almost as great as before; and I asked him whether he had got any facts to justify his observation, but he could give the Committee none. Do you think that it is so?—Yes; we have plenty of cases.

"312. Have you any reason to believe that the normal treatment of lunatics in private asylums, is, that their heads are thumped against the floor, and that they are seized by the throat, as you have described, and so on?—When I used those words, I was referring to the refractory wards of a *county* asylum. I did not say Bethlem; I suspect that that is the normal way of treating them."

Still suspicion alone; nothing but baseless suspicion. Turn for a moment now to Mr. Perceval's opinions respecting the Lunacy Commission and its members, and admit at least the consistency of his assertions on lunacy matters. He speaks of the Commissioners in Lunacy in the following manner :—

"The Board of Commissioners in Lunacy is another inquisition before which the patient has no right to be present, either in person or by attorney; he does not know when the court is summoned, nor what takes place, restoring to us the cruelty of the inquisition, to the mind, and sometimes to the body of the individual."—(Qy. 179.) "It has been a complaint against the Commissioners in Lunacy, that they act with a pettifogging mind on the subject; they have never taken a liberal view of their duties."—(Qy. 243.) "I consider the protection afforded by the visits of the magistrates and Commissioners in Lunacy is very often a mere mockery, aud worse than nothing, because it tempts the patients to complain, and then they are punished vindictively for complaining of anything."—(Qy. 321.) "There is something in the character of the Board of Commissioners in Lunacy that I do not like; I look upon it with suspicion, and I would rather always be under the protection of the magistrates than under the Commissioners in Lunacy. There is a frankness of character in the magistrate, while on the contrary, there is in the Board of Commissioners in Lunacy a duplicity, I rather think; and what is more, although I do not know how to express it, except by the use of a very common word; I will call it an adherence to technicalities."—(Qy. 374.)

We will append here the only quotation it is necessary to make from Admiral Saumarez's evidence to show that his method of assertion was very similar to that of Mr. Perceval. Speaking of the Chancery Court, Admiral Saumarez said :—

"The whole proceedings in Chancery are a sink of iniquity; it is the plunder of the many for the benefit of the few. . . . The Committee were pleased to ask Mr. Barlow how it was that the increase of commissions in lunacy had become so great, there having been eighty last year, and a few years before only forty. I take the liberty to say, that it is from the facility now afforded to designing persons to sue out commissions of lunacy upon parties whom they wish to confine, and whose estates they want to get possession of."—(Qy. 70.)

We wish simply to show the parallelism between the style of assertion of the chairman of the Alleged Lunatics' Friend Society and the honorary secretary of the same society. We shall not have to return to Admiral Saumarez's evidence, as he confined himself almost solely to legal matters, which we should certainly not presume to judge of from the light of his so-called facts.

We might now perhaps leave Mr. Perceval's evidence to the contempt of our readers, and we can fancy that some are indignantly asking why we take upon ourselves the liberty of nauseating them, and upsetting their moral digestion for a brief space with such stuff? The fact is that it is of more importance than at first seems. The public in its notions of lunacy matters errs from ignorance solely. Evidence like that of Mr. Perceval, Admiral Saumarez, and (we regret to class this nobleman in the same category, but facts are relentless) Lord Shaftesbury, coincides with the public notions, and goes beyond them in exaggeration. The public, judging of these gentlemen from the positions they hold, looks upon their opinions as founded on a careful consideration of facts; it is our duty—and an exceedingly important and painful duty—to show that they are the product of a too active imagination only. The interest of the profession, and more particularly that of the unfortunate lunatic, is dependent in no small degree upon the success with which we discharge this duty. We claim the assistance of our readers in executing it; and if it be done well, we have little doubt we shall soon see the *quasi*-philanthropical motives which appear to have actuated these witnesses rightly appreciated.

Before taking leave of Mr. Perceval's evidence, it is but just, however, as a species of corrective to the obnoxious quotations we have given from it, that he should be made to minister occasion for the exercise of the nimbleness of our reader's lungs. This certain of his suggestions for bettering the condition of lunatics are well calculated to do.

"According to my ideas, every asylum ought to be constituted in this way; there should be a governor, as well as a medical superintendent and a clergyman. My reason for stating that is this, that I think patients have sometimes to be protected against medical advice and *experiments*, as well as against any other danger. Another reason is this, that the science of treating the insane is still very imperfect. How far it is proper to admit a minister of religion to interfere with those cases is still very little known, and very much questioned and doubted. Cases might arise where a person, knowing his own constitution and what he had suffered from certain medical treatment, might object to a medical officer's way of medically treating him. He ought to have some one to appeal to to rescue him from it. He might sustain very great injury from it. I know a patient who to the last hour of his life will regret an operation that was performed upon him, by which the temporal artery was severed, and by which he believes that the operations of his mind have ever been in a manner constantly affected; and he does so not only from his own subsequent experience in observing the operations of his mind, but also on a principle that he believes in, that lunacy depends more than anything else upon the state of the blood and the circulation of the blood. I think, therefore, on that account, that every patient should have an opportunity of referring immediately, and appealing to a person such as a governor, against medical treatment which he thought would do him an injury. At present he is like a child; if he will not swallow the dose, it is forced down his throat. I suppose that a governor, in such a case, would either consult the man and respect his experience, or consult his friends to ascertain whether that experience was true, or send for another physician to ascertain what was best to be done. Again, I imagine in the present state of things that there might be improper interference, either on the part of the clergyman with the patient as to his medical state, or by the medical man with the clergyman's interference with the patient from prejudices of his own. I think it is requisite that there should be a governor appointed, a man of liberal education and mind, to whom such cases should be referred for decision, and from which the clergyman might appeal, if he chose, to the Commissioners in Lunacy; but I think it very often might be the case that a medical man would improperly interfere with the clergyman's duties, and also with more probability that the clergyman at this day might interfere injudiciously with the medical man." . .

"231. Mr. *Kendall.* "What class of persons would you select for governors; is he to have a veto in all matters of dispute between the medical officer and the clergyman?—There would be a difficulty, I think, at first; the Government would be compelled perhaps to make the governors of some of our private lunatic doctors; I should not object to that, and for this reason; when you abolish private asylums you abolish the means of living of a great number of men, and I should not like to see that done; at least, it would be very cruel upon them that they should be deprived of those means of living, and I think if they, with their experience, were appointed, in the first instance, as governors, they might act very well as governors, and without suspicion of any misconduct, though as proprietors where their own interests are concerned they are not to be depended upon. *But I do not hesitate to say that I think the persons that ought to be employed by the Government are young men of the aristocracy, and young men of any liberal profession, and I have no doubt that you would find them.* No doubt as years went on, there would be many who would come forward, and that some would be found now. I think it would require men of well-educated, liberal, and delicate minds to undertake these cases; they also must be men of firm temper and resolution; I quite conceive that it would be a difficult thing to get many such men at first, but I think as the subject was discussed, and as men's minds opened to it, what is perhaps ridiculed now, they would really feel it to be an honour and a pleasure to be engaged in, that is in carrying out the benevolent objects of the Government."

As to clerical visitors, Mr. Perceval remarks :—

"Clerical men might see at first, but I think they are entitled to acquire experience in the working of these hospitals as well as medical men. The medical men have been walking the hospitals for years, and practising sufficient cruelty, and causing the death of many patients, and are at last only arriving at a certain amount of knowledge how to treat those disorders. Therefore, I think it no objection to clergymen to say they may err at first in their treatment, and I think that they might be allowed to walk the hospitals and acquire information."*

In his reply to the one hundred and seventieth query, Mr. Perceval gave as one of his reasons for the modifications he suggested, the protection of the patients from medical experiments, and yet he would make the poor lunatic the grouud of a gigantic experiment with unskilled hands!

Finally, Mr. Perceval culminates in the following remarks and reference—

"The great object of all my observations is this—if I can, to break through that constant routine and reference to the medical man; and if I can, to elevate the view which the Legislature should take of this subject, and that they may consider more the spiritual and moral part of the subject than the medical part of it. I find that Lord Shaftesbury states, that only in one out of twenty boards did they find a case in which medical circumstances were combined with the disorder. I think that that is a proof how much the subject should be treated in a moral and a spiritual sense."†

And now to more serious matter again. Mr. Campbell was questioned upon the occurrence of gross acts of cruelty in both public and private asylums (Mr. Perceval having asserted the occurrence of such acts); he was asked if he had any reason to believe that these cases were numerous and frequent. Mr. Campbell answered, "I can hardly say numerous and frequent, but I am certain that they occur at the present time; it is very rare that several months pass by without some cases coming under our notice."‡ This is the reply of one of the Commissioners of Lunacy, and we might reasonably hope now that we have got upon some stable ground. We shall see in the sequel. Mr. Campbell was further asked:—

"404. Can you put in, on another day, the number of the entries as to violence which you or any other Commissioner joined with you in the visitations have made?—I can look over them. I wish to convey to the Committee my impression, that there is truth in the statement that violence is used towards patients by the attendants, and I wish strongly to urge upon them my opinion, that the persons employed as attendants in all asylums, both public and private, are all of too low a class; they are an uneducated class.

"405. You, stating as a public officer that that is your impression, the Committee have a right to know upon what facts it is that you base that opinion; because an impression is good or bad according as you can substantiate it by facts?—The reason why I think that superior persons ought to be employed is, that the duties of an attendant on an insane person are very trying and difficult.

"406. Could you not before the time when the Committee meets again,

* Query 373. † Query 377. ‡ Query 384.

refer to your books, and see whether, not merely as to your inspection, but as to the notice generally of the cases of supposed cruelty which have occurred either in public or private asylums, or in both, and, without enumerating them, give us upon your official authority something like an account of the amount of cruelty which exists in these asylums, and the report you have made in your visiting entries to the authorities of the asylums at the time?—I have no doubt that I shall be able to bring some cases before the Committee; but I am afraid they will appear very small, and hardly to back up what I have stated. I was only stating my impression."

In his evidence on a subsequent day Mr. Campbell produced the evidence for his impression. He said:—

"I was requested to procure some documents in order to show that those acts of cruelty were occasionally committed in asylums; and I have accordingly searched a number of entries, which have been made for some time past; *I may say that we keep no register of those acts of cruelty, which are investigated on the occasion of our visits, and therefore it is with some difficulty that I have got at a few cases.* I have had to go through the entries."*

No register of acts of cruelty! Difficulty of referring to such facts! This seems passing strange. Let us proceed however.

We are told that the cases of complaint of acts of cruelty frequently break down on investigation. Next we are given to understand that only two cases of dismissal of attendants for cruelty could be found to have occurred in county asylums in 1858 and 1859; "but I have noticed in a register that we keep, that a fortnight ago two nurses were discharged from a county asylum in the neighbourhood of London for violence to a patient;"† discharged, that is, without the interference of the Commissioners. What follows of immediate interest to us is too important not to be given in Mr. Campbell's own words:—

"462. From 1847 to 1853, what returns have been made to you of cases of cruelty, and the grounds upon which the allegations have been made?—They have been taken out as they stand in the book, and they are entered in the book according to the alphabetical arrangement of names; and I find that 49 male attendants have been discharged from county asylums for striking and ill-using patients.

* Query 432.

† Query 445.—This is Mr. Campbell's account of the cause of dismissal of these attendants:—"In the month of July, 1858, a patient at a county asylum stated that he had seen two other patients struck with a rope by one of the attendants. That complaint was investigated, I happened to be the Commissioner who visited the asylum. The facts of course were denied by the attendants, but we had them before us, and we came to the conclusion that the facts as stated were true, and that the man had been struck by one of the attendants with a rope. He stated it was a little piece of string which had come out of a bedstead. The patient stated that it was a heavy rope, and the result was that this attendant and another who was implicated were discharged on that occasion. We made an entry of this case at the time, and although the medical superintendent of the asylum stated that the men bore very good characters, they were discharged." This is an example of *cruelty*.

"463. That is to say, the superintendents of the county asylums, or the committee of management, have dismissed those attendants? —Yes, they are cases which have been investigated and decided upon. 464. They are cases in which the superintendents of the asylums, or the committee of management, have dismissed the attendants for not discharging their duty?—Yes; that is, in which they found that the attendants had struck or injured patients. 465. Mr. *Briscoe.* Or otherwise ill-used them?—Yes. 466. Sir *George Grey.* The words in the Act are, 'Dismissal for misconduct of any nurse or attendant'? —Yes. 467. Do you include misconduct of every kind that has led to the dismissal of an attendant?—No; I have only directed such cases to be extracted as show real cruelty, in order to bear out what I stated on a former occasion. 468. In addition to those cases, do you believe that there are numerous other cases in which attendants have been dismissed for misconduct of another kind?—I know that there are a great many; I cannot say how many. 469. Are not those cases equally reported to the Commissioners under the terms of the Act of Parliament? Yes, and they are in the book; but I have not looked to see how many. 470. Are they numerous?—Yes; I may mention that not even cases of neglect are set down in this list. . . . .

"480. Then the proportion of cases in which alleged cruelty was complained of have been, in the course of twelve years, forty-nine in 1000 or 1500 attendants?—Forty-nine men and twenty-five women; but I should remind the Committee, that these are cases which have not been complaints only, but cases which have been proved and followed up by dismissal, and in some cases by prosecutions, and fine and imprisonment. 481. Colonel *Clifford.* Have you not returns for two years which are more close?—I find that of those forty-nine male attendants who were discharged, ten were discharged in the years 1858 and 1859; and of the women, five were discharged within the same period. 482. Mr. *Henley.* Can you state how many were discharged in consequence of the recommendations of the Commissioners, as compared with those who were discharged by the managers of the asylums?—I have no means of knowing, but I suspect that these have been all discharged independently of the Commissioners. 483. If that be so, have you any reason to doubt that, as far as can be, a remedy is immediately applied in those cases where cruelties occur in these public asylums?—Where it is proved, I have not the slightest doubt that punishment immediately follows; I have no doubt that the managers of the public asylums at once dismiss the attendants who have been proved to have committed any act of cruelty. 484. Mr. *Tite.* Does that answer apply to private asylums as well as public asylums?—I think so. 485. As much to one as to the other?—I think it does. . . . .

"502. Have you anything to add that will lead to the conclusion that there are more cases than those which appear to have been reported to your office under the directions of the Act of Parliament?—I have before stated that these cases do not include cases of gross neglect, or any minor instances of cruelty. 503. Will you be good enough to answer my question?—I have no more that I can bring forward. 504.

I am not inquiring into any cases of neglect; I wish to confine my inquiry to cases which come under the description of cruelty.—That has been strictly attended to in the lists which have been prepared. 505. You cannot add any facts but those which you have already stated?—No. 506. *Chairman.* Will you now, if you please, go to the private asylums?—The complaints in licensed houses, which I shall now mention, have occurred within the last two years, 1858 and 1859, and there are only four of them. 507. Those are cases which have been brought under the notice of the Commissioners?—Yes; in the metropolitan licensed houses. 508. Are they cases of dismissal?—In some cases, of dismissal. 509. Are these only reports of cases that you have received during the last two years?—No; these cases came under our own knowledge. 510. How many cases of alleged cruelty have been reported to you during the last two years in private asylums?—Nine males and one female in 1858 and 1859. 511. Can you give a comparison between 1847 and 1858?—The total was 48 male attendants in licensed houses discharged for violence towards the patients, and 20 females. 512. *Chairman.* Making together 68?—Yes. 513. *Sir Erskine Perry.* Does that number contain all: I understood you to say that you did not get all the returns?—I can only say that we sent the circular to which I have referred some time ago. . . . .

"519. Were those persons discharged in consequence of reports made by the Commissioners, or were they discharged by the proprietors of the asylums?—I should say that they had all been discharged by the proprietors of the licensed houses. 520. Then with regard to the management of asylums, with reference to the cases of dismissal for cruelty, the private and public asylums seem to be pretty much upon a par?—It seems so by the return. 521. Have you any reason to believe, either as to the private or public asylums, that the managers of either one or the other do not take pains to dismiss attendants, either male or female, in cases of cruelty which are brought to their notice?—No; I have no reason to believe that. 522. Have you any reason to believe that they do not take sufficient pains to ascertain that the cases of cruelty are properly inquired into as soon as they occur?—No; I think all proprietors of asylums, as well as superintendents of asylums, are anxious to get the best attendants that they can. 523. Have you in your visits to asylums, whether public or private, heard any complaints made on the part of patients, that cases of cruelty have been brought to the notice of the superintendents, proprietors, or managers of those asylums, which cases have not been attended to?—Yes; I think I may say that that has been the case. 524. To any great extent?—No; a patient will come and say, 'I complained that such and such a nurse had been rough with me, but Mr. So-and-so would not believe me.' I have then seen the nurse, and I have said, 'Is that true?' and, as a matter of course, she has said that it was not true. 525. You have no reason to believe that cases of cruelty occur and are complained of, without their being attended to on the part of those who ought to take notice of them?—No, I have no reason to believe that. . . . .

"531. Mr. *Henley.* Do you think that the fact of the number of the complaints being sixty-eight in the private houses, and seventy-four in the course of twelve years in the public asylums, bears out the former part of your evidence, that you thought cruelty was especially practised in county asylums?—With reference to that, I beg to state that I contradicted myself, when I said especially; I believe I intimated to the Committee at the time that I did not mean especially in public asylums. 532. You stated in your former evidence that you agreed with what Mr. Perceval had stated in many things; do you wish to amend that part of your evidence?—I wish to take away the words 'especially in public asylums,' because I do not mean that. 533. Again, at Question 385, in answer to this question by me, 'You have stated that it is especially so in public asylums;' your answer is, 'I think, perhaps, I may say so.' Having given that opinion in two cases, do you wish to adhere to it?—I think I would amend it in this way; I may say in some of the refractory wards of county asylums."

If we turn now to the Appendix of the Report, to see in what manner Mr. Campbell's returns of cases of *cruelty* bear out his impressions with regard to the occurrence of such cruelty, not forgetting that gentleman's assertions that he had "only directed such cases to be extracted as show *real cruelty*," we find, first, that the whole of the returns of dismissals of attendants are simply headed for "*ill-treatment* of patients." Again, of the 142 cases of dismissal recorded, forty-four were for "striking a patient;" one for returning a blow; one for impertinence to a patient; against two attendants only does it seem to have been necessary to proceed at law; one attendant was dismissed for maltreating a patient, though in a trifling manner; in three cases only does there appear upon the face of the return *cruelty*, properly so called; and all the rest were cases of dismissal for various degrees of ill-usage of, or harshness towards patients.

It is evident, therefore, that if Mr. Campbell's use of the word *cruelty* is to be received in the ordinary signification of that word, "inhumanity, savageness, barbarity," his returns bear out his assertions very imperfectly. And thus it would appear that whether we take these returns at that gentleman's estimate, or if we take them at our own estimate, as obtained from the returns themselves, the charge of cruelty in asylums, as received, approved of, and expressed by this representative of the Lunacy Commission, is baseless. All the facts go to prove that, under present circumstances, instances of cruelty in our asylums are rare, and that the Commissioners of Lunacy are in a position to prove this.

It is much to be regretted that gentlemen occupying highly responsible official positions should make serious charges against a respectable body of men, without being able to support such imputations by a reference to specific facts.

Dr. Bucknill put the question of so-called cruelty on its right foundation when he told the Committee, speaking of public asylums, that he believed "cases of deliberate cruelty are exceedingly rare; but the stress upon the temper and patience of the attendants is so great that loss of temper is not uncommon."* That stress will always remain unless automaton attendants can be invented, and the evils naturally resulting from it can only be met by strict supervision, and a large number of attendants. Mr. Campbell's own statements show how strict is the discipline kept both in public and private asylums. Immediate dismissal is the punishment of *any* violence or harshness towards a patient, except it be in the most manifest self-defence. This is the only plea allowed in exculpation, and so efficiently do the superintendents of public asylums, and the proprietors of private, carry this rule into action, that the Commissioners have very rarely to interfere.

We now come to the suggestions offered to the Committee.

First in importance are those of the Commissioners in Lunacy, contained in a memorandum printed in the Appendix to the Report.† These, however, although of most moment, require only to be briefly glanced at, since they formed the basis of the suggestions made by Lord Shaftesbury, and we noted their general character and objectionable points in reviewing the propositions contained in the proposed Bills discussed by the Committee of 1858-59. The Commissioners' suggestions contain the following provisions:—1. For an increased number of visits of the Commissioners and visitors to licensed houses during the year. Every house to be visited regularly six times a year by the visitors, and three times at least by the Commissioners, "altogether at least nine times, or, on the average, once in six weeks." 2. The Commissioners to be empowered to demand information as to the payments and expenditure for private patients.—No facts have been laid before the Committee warranting such a provision; it is based almost entirely upon an unjustifiable suspicion; and it is not needed, as the following extract from a letter of the Secretary of the Commissioners to the Lord Chancellor will show:—

"In the course of their visits, the Commissioners have from time to time had great reason to believe that the patients did not derive such comforts and accommodation from their income and allowance as they were entitled to, and they have, therefore, in many cases, required from the persons in whose care patients have been placed, and others, a statement of the patient's property, and of the sums

* Query 1008. † P. 209.

disbursed on their account. *This information has generally been very readily given*, and for the most part with beneficial results."*

Just so; give the Commissioners, if necessary, powers by which they could obtain the information required by the aid of the magistrates or some other tribunal, *upon due and sufficient cause being shown;* but to give them a general and unlimited power to obtain information which is already willingly given in most instances, would be a piece of the most vexatious legislation. The proposition is not a *boná-fide* one, and, supposing it enacted, if it were carried out in the spirit indicated in the evidence of the chairman of the Lunacy Board, it would probably lead to endless interference as to rates of payments, since the opinions of the members of the Board would become the standard of reference, not the professional character or position of the medical man to whose care the patient was consigned. — 3. More careful provisions respecting the signing of certificates for admission into asylums by relatives; for the discharge of patients; and for amendment of informal certificates. 4. Provisions for the better medical charge of licensed houses; every house for 50 patients to have a resident medical proprietor or medical officer; every house for 30 patients and less than 50, to be visited daily by a medical man, if one be not resident; every house for less than 30 to be visited by a medical man thrice a week, unless in cases of dispensation by the Commissioners or visitors. 5. Copies of Commissioners' entries to be sent to visitors; copies of both Commissioners' and visitors' entries to be laid before justices in Quarter Session. 6. Provision for discharging patients by Commissioners in certain special cases. 7. Provision by which Commissioners could grant leave of absence "on trial," from an asylum to a patient. 8. Provisions for the registration of persons who take single patients under care, the Commissioners also to have the power to call "for any particulars relative to single patients or their property and payments which they may think fit." The provision not to extend to persons residing with relatives, no one of whom, directly or indirectly, receives payments for the charge.—The unlimited power to demand information as to payments of single patients is open to the same objections as that referring to patients in licensed houses.—9. Provision that the definition of physician, surgeon, apothecary be conformable to the Medical Act. 10. Certain provisions facilitating arrangements for providing additional accommodation, whether temporary or not, and for obtaining burial grounds in connexion with public asylums.

Mr. Campbell laid before the Committee a separate memorandum† of suggestions, of which we may note the follow-

* P. 214. † P. 195.

ing particulars:—1. Every order, statement, and medical certificate, to be written in duplicate by the persons signing the same. One of the copies to be sent to the office of the Commissioners in Lunacy, instead of the copy now required to be made by the proprietor or superintendent. 2. Any one Commissioner to be empowered (in addition to the ordinary statutory visits by two Commissioners) to visit at his discretion any asylum, licensed house, hospital or gaol, and make such inquiries as he may think fit. "Give unlimited powers of inquiry, but add some of the most material matters, including for payments and dietary of all classes."—Is not this highly significant of the mode in which the unlimited power sought by the Commissioners would be carried out?—3.—16 and 17 Vict. c. 96—"Provide that persons signing order, shall have seen patient within one week of signing such order, and have satisfied himself that the patient is insane; that he or some person deputed by him should visit patient within three months after first admission, and subsequently every six months. Medical statement accompanying the notice of admission to be much more specific, and give 'facts or symptoms' showing insanity. Empower Commissioners in all doubtful cases to call for second statement within a month." 4. "Require a report from medical proprietor or medical superintendent or attendant after three weeks, and within four weeks, after admission of a patient, in such form as it by order may require. This to be in addition to the full statement required with the notice of admission. Add a penalty for failure. Where, upon such second report, the insanity of the patient appears doubtful, he shall be visited specially, within seven days, by a Commissioner or the medical visitor (in the case of a provincial licensed house), or (in the case of a hospital) by two members of the Committee, or (in the case of a metropolitan house) by a Commissioner."

Mr. Purnell Bransby Purnell, Visitor of the several lunatic asylums of the county of Gloucester, laid before the Committee a paper containing his views respecting the Law of Lunacy as it referred to magisterial districts. We commend Mr. Purnell's paper to the notice of our readers, not that we coincide with many of its details, but because it bespeaks a careful and thoughtful consideration of the subject on which he writes. One remark of this gentleman, referring to magistrates, may be especially noted as having a much wider application. He says:— "*It becomes a mistake in legislation, as in the sections alluded to, by abridging their powers and duties, and transferring them to the Commissioners, to show a distrust either of the ability or fidelity with which they would exercise and perform them, the result being that magistrates the best qualified for*

*visiting might decline to act.*" Mr. Purnell has, we think, overlooked the principle contained in this remark in his depreciatory observations on apothecaries, which observations are evidently founded on some erroneous conception of the qualifications of the apothecary, properly so called.

Of the suggestions and suggestive information scattered through the evidence, we may notice propositions for a greater freedom of correspondence by lunatics in asylums, and greater facilities of admission to friends. These are points with regard to which no definite rules can be laid down, as they must in the end be left to the decision of the medical man in charge, the friends, and the Commissioners. The Commissioners urge that higher rates of wages should be given to attendants in asylums, hoping thereby to attract a better class of persons to such posts. Dr. Bucknill doubts whether a better class could be obtained than now, and asserts that it is an increased number of attendants that is wanted. The instances of harshness now occurring from time to time towards patients, depend more upon the stress of duty upon the attendants from being insufficient in number, than upon their personal character. Dr. Coxe, one of the Medical Commissioners of the Scotch Lunacy Board, modifies our ideas somewhat respecting the working of the chartered asylums of that division of the kingdom. He tells us that on the whole the system works very well; but he thinks it a bad plan when the pauper and wealthier patients are kept under the same roof. He approves of the system most when the two classes are kept in different buildings—when, in fact, they are provided for in different, although it may be contiguous, asylums. He tells us also that the treatment is regulated by the payment,* and that there is a considerable saving from the rate of maintenance paid for the private patients, which goes not so much to increase the comforts of the pauper patients, as to extend the institution.† Other, to us, rather serious objections, also are made known in Dr. Coxe's evidence, to which we would refer our readers on account of its quiet, substantial value. We so recently gave an analysis of the First Annual Report of the Scotch Lunacy Board, that we need not dwell any longer upon this evidence. Hitherto we have not alluded to the evidence given by Mr. A. Doyle, one of the Inspectors under the Poor-Law Board. This is, indeed, so important in regard to pauper lunacy that we have noticed it apart.‡

Before closing this abstract of the suggestions, we would briefly refer to one made by Dr. Bucknill—to wit, that an asylum should be established for the *testing* of doubtful cases of lunacy. This, to be termed a *Probationary Asylum*, he proposes to put under the

* Qy. 1414. † Qy. 1324. ‡ See page 109.

sole charge of the Commissioners in Lunacy, and that it should be available for all parts of the kingdom. The cases to be admitted are those which have already been sent to some asylum, but upon whose insanity doubt rests. Even on the supposition that this would be a feasible way of getting over the difficulty named, it would be necessary first to show that the difficulty really existed to an extent to warrant a special interference. No such necessity was, however, shown, and we regret that, by an ill-considered and gratuitous project, a portion of the medical evidence given before the Committee should have been made to approximate, however slightly, in laxity to some of the lay-evidence which we have had so strongly to condemn.

We have purposely avoided making any observations on the lay-evidence as it bears upon Chancery lunatics. The wild speculation thrown over that branch of the inquiry with which we are most familiar warns us against treading upon ground with which we have a slighter acquaintance.

Our examination of the evidence laid before the Committee of last session is now ended. The *first* conclusion to which we have been led by that examination has been this:—That the popular outcry which led to the parliamentary inquiry has been in a great measure devoid of foundation. The *second*, that, in so far as asylums are concerned, the majority of the suggestions brought forward for the amendment of the law, are based upon surmise, and not upon fact. The *third*, that, if legislation to any extent be carried out upon the said suggestions, it will simply complicate still more an already sufficiently complicated subject; it will, in fact, be vexatious, and would probably tend to increase the very evils it sought to amend.

Further, we doubt whether any of the propositions submitted to the Committee (except in relation to workhouses) will add to the protection already afforded to the lunatic, either before his admission into an asylum or after. It is not to increased legislation that we must look most for increased protection. The law gives the Commissioners in Lunacy even now more power for the care of the lunatic than they feel justified in using.* Let the public be disabused of the erroneous notions it entertains respecting the treatment of lunatics, and it would soon perceive that under the care of the Lunacy Commissioners such evils as still beset the proper management of the insane are being gradually but steadily done away with, and that further improvement is to be attained rather by a right accord being kept between public feeling and the Commissioners on the one hand, and the Commissioners and the medical profession and proprietors of

* See 13th Report of Commissioners, p. 52.

asylums on the other, than by legislation. The present popular outcry, in whatever manner it may have been apparently justified at the commencement, cannot be supported by any sufficient series of facts, upon the showing of the Commissioners; but being made by them a plea for further legislation, it must almost necessarily lead to harm, not solely by occasioning the enaction of unnecessary laws, but by directing public thought into a false channel.

Again, the grave, yet unsustained charges made by the Chairman of the Lunacy Commission, and not dissented from by the other Commissioners examined, against the medical profession practising in lunacy and the proprietors of private asylums, must tend to interfere with that cordial co-operation between the one and the other which is so important for the proper carrying out of the delicate and responsible duties of the Commission.

We might justly complain of the tone adopted by the official witnesses towards the medical profession in the course of this inquiry; we might rightly ask, how it happens that a profession which has lavished so much labour and pains upon the care of the lunatic, which has withheld none of its experience from the public, and to which the public is indebted for all the knowledge by which the condition of the insane has been ameliorated, is at this day exposed to wanton charges, touching nearly its moral character? But we forbear: we shall be content if we can disabuse the public mind of a portion of the errors which it entertains concerning the treatment of lunatics, and facilitate in some degree a clearer knowledge of what is required for further benefiting them.

We, however, protest against the persistent attempts made in the present inquiry by many of the witnesses to elevate the so-called civil rights of the lunatic above his rights as a sick man. Is the nascent lunatic to be matured into a perfect one, in order that a few individuals may parade, under a pseudo-philanthropic guise, their care for the "rights of the subject"? Has not the ailing subject a right to be cured, and is not this his primary right? But we have not a word as yet on the important question how this right is to be secured in the early stages of insanity, without needlessly affecting the liberty of the individual, or stamping him prematurely as a lunatic. Put it aside as we may, this is the great question in lunacy legislation. To set it rightly to rest would require a liberal appreciation, on the part of the people, of the duties of the medical man; and we greatly fear that the unfortunate tone assumed by the official witnesses in the present inquiry, will blast any hopes of this feeling being brought about rapidly among the public.

